# miRNA-425-5p enhances lung cancer growth via the PTEN/PI3K/AKT signaling axis

**DOI:** 10.1186/s12890-020-01261-0

**Published:** 2020-08-24

**Authors:** Jin-shan Zhou, Ze-shan Yang, Si-yang Cheng, Jiang-hao Yu, Chao-Jun Huang, Qiang Feng

**Affiliations:** Cardiothoracic Surgery, The Fourth Affiliated Hospital, Zhejiang University School of Medicine, Shangchen Road NO.1 of Yiwu, Zhejiang, 322000 China

**Keywords:** miR-425-5p, Lung cancer, PTEN, PI3K/AKT signaling pathways

## Abstract

**Background:**

miRNAs regulate a multitude of cellular processes and their aberrant regulation is linked to human cancer. However, the role of miR-425-5p in lung cancer (LCa) is still largely unclear. Here, we explored the role of miR-425-5p during LCa tumorigenesis.

**Methods:**

Cell proliferation was evaluated by cell counting Kit-8 and colony formation assay. Western blot and real-time PCR were accordingly used to detect the relevant proteins, miRNA and gene expression. Luciferase reporter assays were used to illustrate the interaction between miR-425-5p and PTEN.

**Results:**

We demonstrate that miR-425-5p is overexpressed in LCa tissue and enhances the proliferative and colony formation capacity of the LCa cell lines A549 and NCI-H1299. Through predictive binding assays, PTEN was identified as a direct gene target and its exogenous expression inhibited the pro-cancer effects of miR-425-5p. Through its ability to down-regulate PTEN, miR-425-5p activated the PI3K/AKT axis.

**Conclusion:**

We conclude that miR-425-5p promotes LCa tumorigenesis through PTEN/PI3K/AKT signaling.

## Background

Lung cancer (LCa) is a leading cause of cancer related mortality across the globe. LCa is prevalent in males [1] and asymptomatic during early disease stages. As many as 2 in every 3 cases are at an advanced stage (III or IV) when diagnosed and the 5-year survival rates remain low, particularly for those with metastatic LCa [2]. Improved LCa therapeutics is thus urgently required.

MicroRNAs (miRNAs) regulate many cell processes including differentiation, metabolism and tumorigenesis [3–6]. Emerging evidence suggests that miRNAs are key players during LCa tumorigenesis [7–10]. The aberrant expression of miR-425-5p is linked to hepatocellular carcinoma (HCC), gastric cancer (GCa) and colorectal cancer (CRC) [11–13]. Here, we report the upregulation of miR-425-5p in LCa and highlight its contribution to LCa development. We further identify PTEN as a novel miR-425-5p target that is inhibited in LCa to promote PTEN/PI3K/AKT signaling.

## Methods

### Patient specimens

LCa samples (*n* = 25) and adjacent healthy tissue (at least 2 cm from the resection margin) were collected from the Fourth Affiliated Hospital, Zhejiang University School of Medicine. The study was fully supported by the Institutional Review Board of the Fourth Affiliated Hospital, Zhejiang University School of Medicine (No.2015-0-09). All participants provided consent for sample analysis and anything about their identities will not be included in the data.

### LCa cell lines, cell culture and cell transfection

Human lung cancer cell lines A549, NCI-H1299, NCI-H460, HCC827 and Normal Human Lung Epithelial cell line (BEAS-2B) were obtained from the Cell Bank of the Chinese Academy of Sciences (Shanghai, China). A549 & HCC827 cells were cultured in DMEM plus 10% FBS and pen/strep. NCI-H1299 & NCI-H460 cells were grown in RPMI-1640 plus 10% FBS at 37 °C in 5% CO_2_. BEAS-2Bs were cultured in Clonetics™ media.

The miR-425-5p mimics and negative control miRNA (NC) were chemically synthesized by Shanghai GenePharma Co., Ltd. (Songjiang, Shanghai, China). Lipofectamine 2000 (Invitrogen, Eugene, OR, USA) was used for transfection according to the manufacturer’s protocol. PI3K activity was assayed as previously described [6]. PI3K inhibitor LY294002 was obtained from Abcam. To analyze the effects of miR-425-5p on PI3K/AKT, indicated Lca cells were cultured in the presence or absence of the drugs for 24 h at 37 °C, the working concentrations of LY294002 were 30 μM. For experiments with LY294002 treatments, indicated Lca cells were pre-treated with LY294002 for 1 h prior to exposure to proteasome inhibitors.

### Cell growth assays

LCa viability was assessed using Cell Counting Kit-8 from Shanghai Haling Biotechnology, Co., Ltd. (Shanghai, China) as per the manufacturer’s protocols. Briefly, after incubating the transfected cells for one full day, they were collected after trypsinization and seeded (~ 5000 cells/well) into 96-well plates. Ten microliters of CCK-8 solution were added per well and kept for 2 h at 37 °C. The absorbance of the mixture was estimated in a microplate reader from Bio-Rad Laboratories, Inc. (Hercules, USA) at 450 nm.

### Colony formation assay

The colony formation assays were performed as previous [6]. Each group of treated cells (1 × 10^3^ per well) was seeded into 10 cm culture dish, and cultured for 2 weeks. Finally, colonies were stained using 1% crystal violet and the number of cell colonies was counted.

### qRT-PCR analysis

Total RNA was isolated by TRIzol® reagent from Invitrogen (Thermo Fisher Scientific, Inc.), and a NanoDrop (NanoDrop Technologies; Thermo Fisher Scientific, Inc.) was used to estimate its quality and concentration. The expression of miR-425-5p was done by reverse transcription using the Mir-X™ miRNA First-Strand Synthesis Kit from TaKaRa Biotechnology, Co., Ltd. (Dalian, China), and quantitative evaluation of the synthesized cDNA was done by quantitative PCR (RT-qPCR) using the Mir-X™ miRNA qRT-PCR TB Green® Kit from TaKaRa Biotechnology. As an endogenous control, the small nuclear RNA U6 normalized the expression of miR-425-5p. The 2^−ΔΔCq^ system was used to evaluate all genes expressions and the primer sequences were shown as follows: miR-425-5p (forward: 5′- GGGGAGTTAGGATTAGGTC-3′, reverse: 5′- TGCGTGTCGTGGAGTC-3′), U6 (forward: 5′-CTCGCT TCGGCAGCACA-3′, reverse: 5′-AAC GCT TCA CGA ATT TGC GT-3′), PTEN (forward: 5′- TGGATTCGACTTAGACTTGACC − 3′, reverse: 5′- AGGATATTGTGCAACTCTGCAA -3′), GAPDH (forward: 5′-CATCACCATCTTCCAGGAGCG-3′, reverse: 5’TGACCTTGCCCACAGCCTT-3′).

### Dual luciferase assays

The design and synthesis of PTEN fragments containing binding sites for WT (wild-type) and MUT (mutant) on miR-425-5p was done by Shanghai GenePharma. These were cloned into the Target Expression Vector pmirGLO Dual-luciferase from Promega Corporation (WI, USA) to get the reporter plasmids WT-PTEN and MUT-PTEN. One night prior to transfection, seeding of cells (60–70% confluence) was done in plates with 24-wells. Transfection of these cells was done with reporter plasmids harboring WT or MUT in the presence of miR-NC or miR-425-5p mimic. Post 48 h of transfection, luciferase activity of the cells was estimated as per instructions using the Dual-Luciferase Reporter Assay System from Promega Corporation (Promega, Fitchburg, WI, USA). The data normalization was done by the activity of Renilla luciferase.

### WB

Cell lysates (RIPA lysates) were resolved on SDS page and transferred to PVDF membranes. Membranes were blocked for 1 h in 5% milk plus TBST, incubated with primary antibodies against PTEN (dilution, 1:5000; cat. no. ab170941; Abcam), PI3K (dilution, 1:8000; cat. no. ab32089; Abcam), p-AKT (dilution, 1:8000; cat. no. ab81283; Abcam), AKT (dilution, 1:8000; cat. no. ab32505; Abcam), p-AKT (dilution, 1:8000; cat. no. ab81283; Abcam), ACTIN (dilution, 1:8000; cat. no. ab8226; Abcam) at 4 °C overnights, and labeled with HRP-conjugated secondary’s for 2 h at room temperature. Bands were visualized using chemiluminescent HRP Substrate and analyzed using Image Lab TM Software.

### Statistical analyses

Data analysis was performed using SPSS 19.0. Treatment groups were compared using a one-way ANOVA. *P*-values < 0.05 were taken as significant. Experiments were performed on at least three occasions. Data represent the mean ± SD.

## Results

### miR-425-5p is upregulated in LCa

**Fig. 1:**
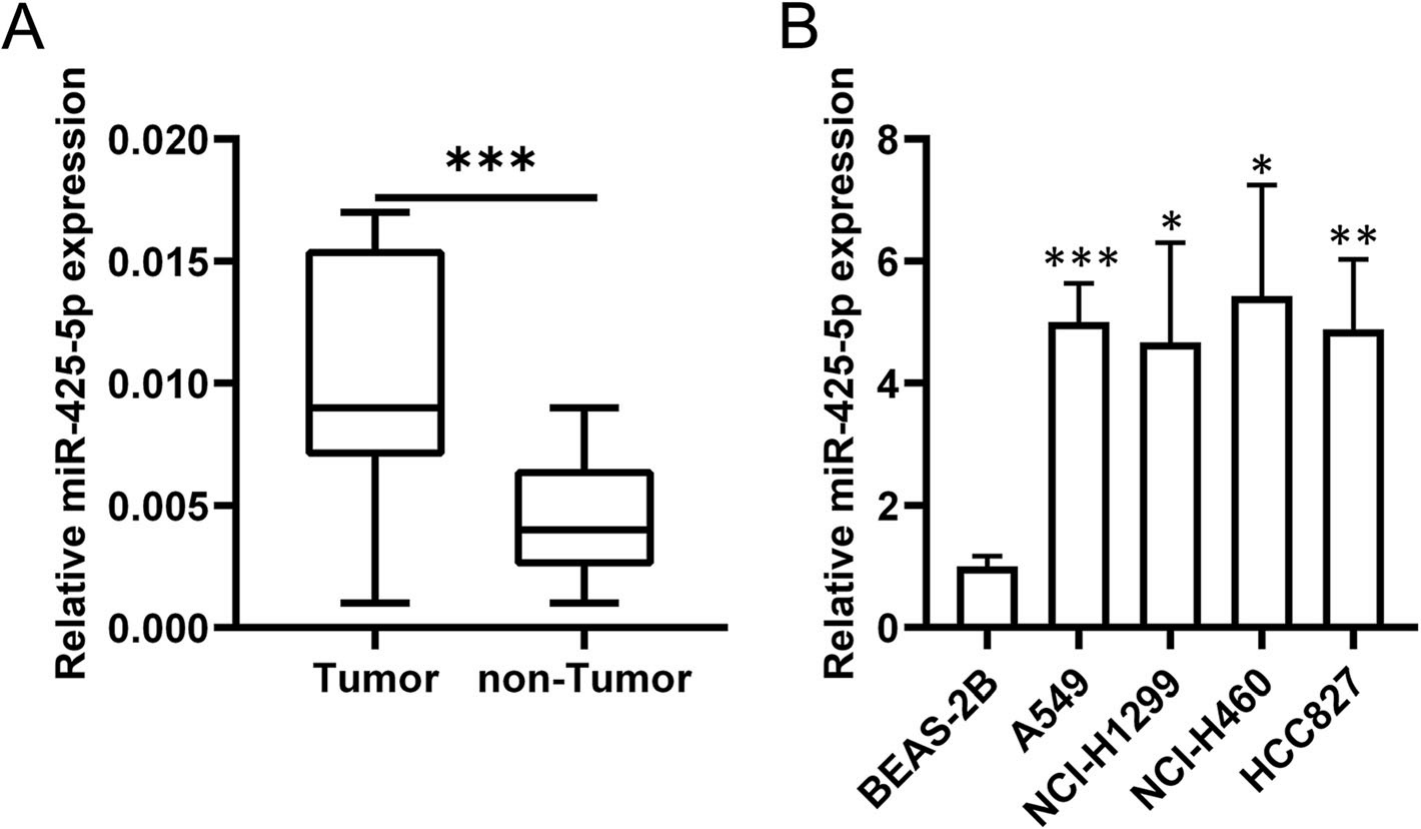
MiR-425-5p in highly expressed in LCa (**a**). qRT-PCR of miR-425-5p. **b**. qRT-PCR of A549, NCI-H1299, NCI-H460, HCC827 and BEAS-2B cells. Data: mean ± SD. * *P* < 0.05; ** *P* < 0.01; *** *P* < 0.001

We compared miR-425-5p levels in 25 paired LCa and normal lung tissue samples by qRT-PCR analysis. miR-425-5p was upregulated in LCa specimens (Fig. 1a) and expressed to high levels in A549, NCI-H1299, NCI-H460, and HCC827 cells compared to normal human lung epithelial cell line (BEAS-2B) (Fig. 1b) consistent with previous findings in other cancer types. Previous results indicated that miR-425-5p is up-regulated in LCa.

### miR-425-5p enhances proliferation and inhibits apoptosis in LCa cells

**Fig. 2:**
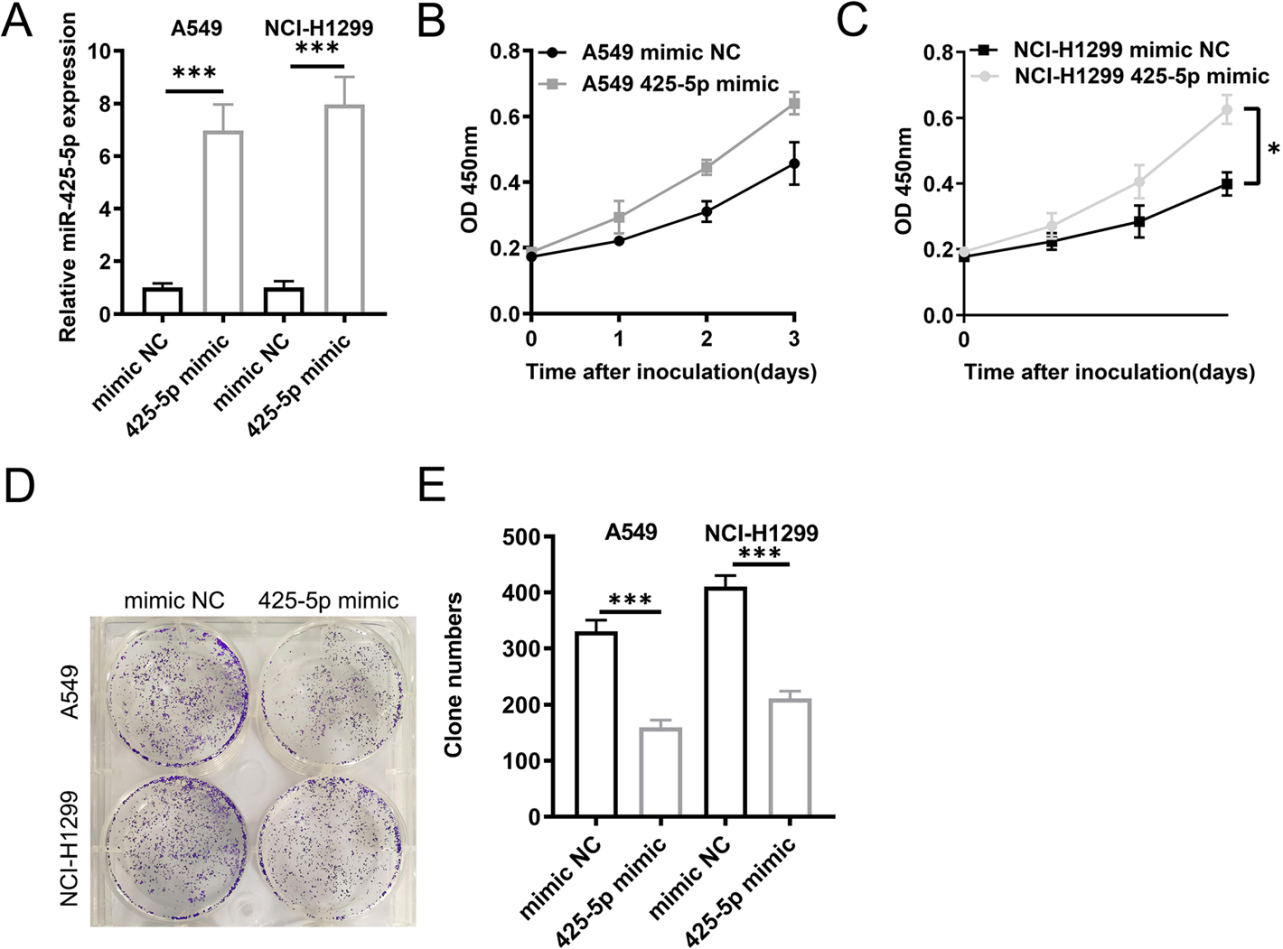
MiR-425-5p promotes LCa. (**a**). miR-425-5p expression in A549 and NCI-H1299 cells. **b** & **c**. CCK-8 assays in miR-425-5p mimic transfected cells. **d** & **e**. Colony-forming assay in miR-425-5p mimic transfected cells. The raw data of all colony formation experiments was listed in supplemental Table 1. Data: mean ± SD. * P < 0.05; ** P < 0.01; *** P < 0.001

To dissect the role of miR-425-5p in LCa, its expression was manipulated using miR-425-5p mimics. Figure 2a-c shows that miR-425-5p upregulation enhanced cell survival, meanwhile enhanced cell colony formation ability (Fig. 2d and e). Taken together, above results indicated miR-425-5p is thus an oncogene in LCa cells.

### miR-425-5p targets PTEN

**Fig. 3:**
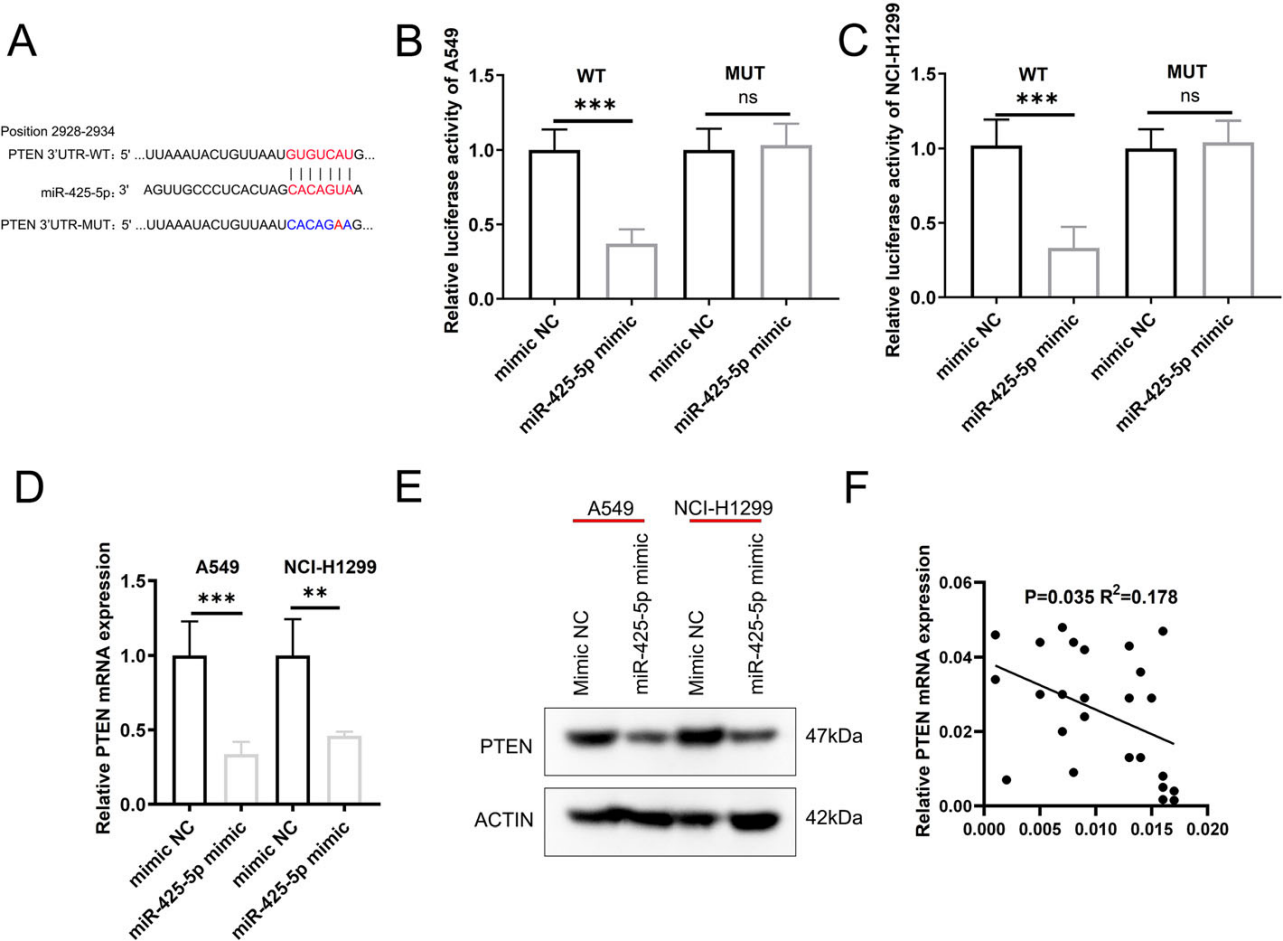
MiR-425-5p targets PTEN in LCa. **a**. Predicted binding of miR-425-5p and PTEN. **b** & **c**. Relative luciferase activity of PTEN-WT, PTEN-MUT in LCa cells expressing miR-425-5p mimic. **d**. PTEN mRNA levels are significantly lower after transfection with miR-425-5p mimic. **e**. PTEN expression assessed by WB. Full-length blots/gels are presented in Supplementary Figure 1. **f**. miR-425-5p and PTEN negatively correlate in LCa tissue. Data: mean ± SD. * P < 0.05; ** P < 0.01; *** P < 0.001

From TargetScan 7, PTEN was identified as a predicted miR-425-5p target (Fig. 3a). In PTEN 3′-UTR reporter assays, miR-425-5p suppressed WT PTEN expression (Fig. 3b-c) but had little effect on mutated PTEN 3′-UTR fragments (Fig. 3b-c). The levels of PTEN were lower in cells transfected with miR-425-5p mimics (Fig. 3d-e). MiR-425-5p also negatively related PTEN mRNA levels in LCa tissue (*P* = 0.035, R^2^ = 0.178, Fig. 3f). These data implicate PTEN as a cellular target of miR-425-5p.

### miR-425-5p promotes LCa via PTEN

**Fig. 4:**
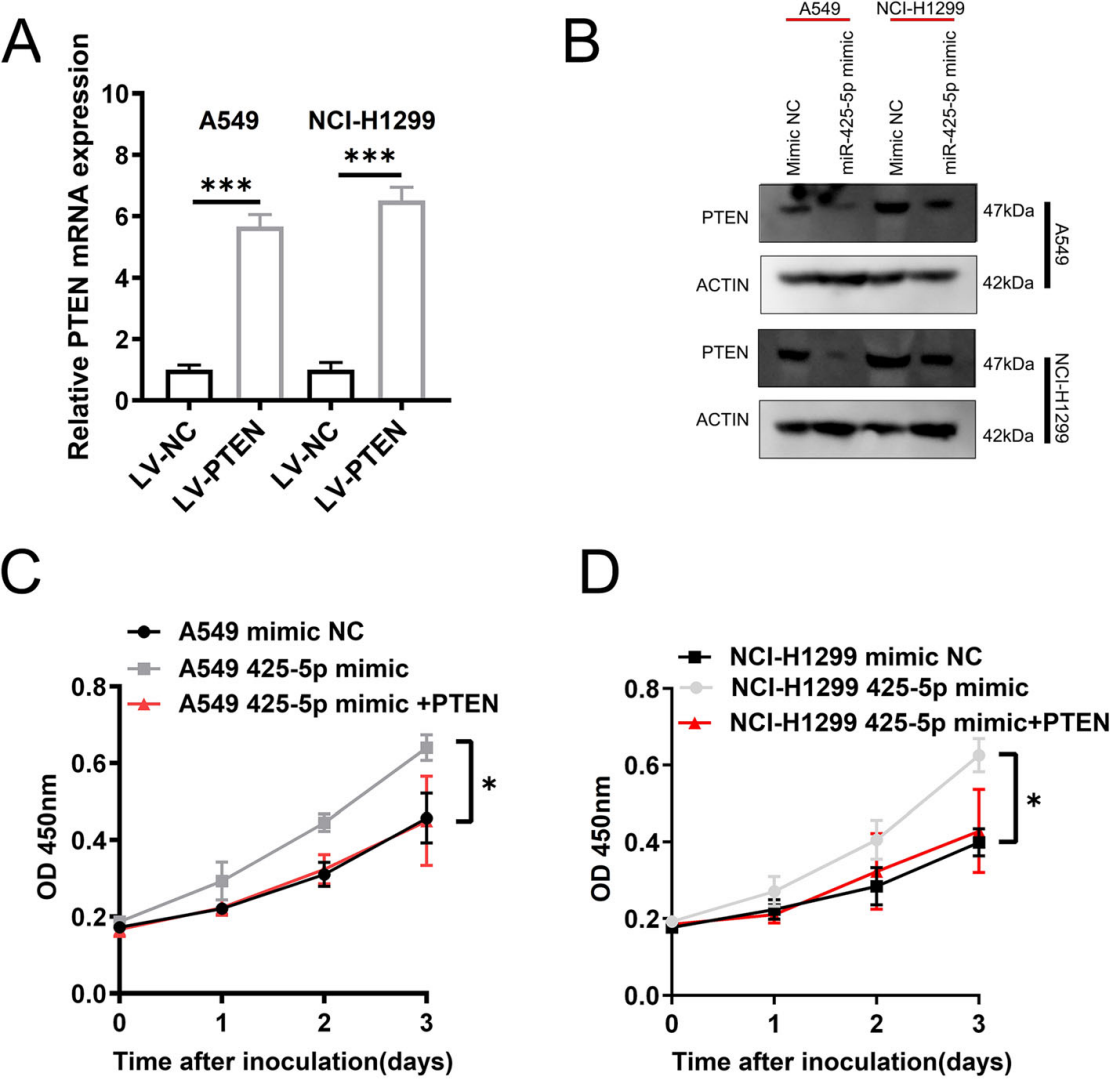
MiR-425-5p promotes LCa growth by targeting PTEN. **a**. qRT-PCR of PTEN in indicated Lca cell lines. **b**. WB analysis of PTEN expression. Full-length blots/gels are presented in Supplementary Figure 2. **c** & **d**. CCK-8 analysis of cell viability in the indicated cell lines with empty vector, miR-425-5p mimic, and/or PTEN in A549/NCI-H1299 cells. Data: mean ± SD. * P < 0.05; ** P < 0.01; *** P < 0.001

To define whether miR-425-5p regulate PTEN in Lca, we firstly overexpression PTEN in LCa (Fig. 4a). WB analysis demonstrated that miR-425-5p reduces PTEN levels which could be recovered by exogenous expression (Fig. 4b). Cell viability assays showed that miR-425-5p enhances LCa proliferation which could be reversed by PTEN transfection (Fig. 4c-d), suggesting miR-425-5p mediates its activities via PTEN. This indicates that miR-425-5p targets PTEN to mediate its pro-tumor effects.

### miR-425-5p activates PTEN/PI3K/AKT signaling

**Fig. 5:**
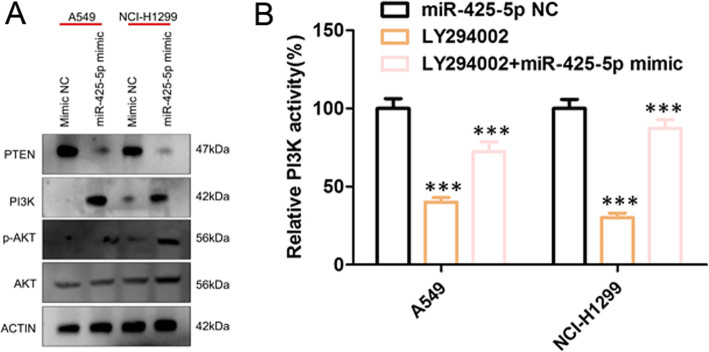
Effects of miR-425-5p on PTEN/PI3K/AKT. **a**. WB analysis of PTEN, PI3K, p-AKT, and AKT in LCa cells. Full-length blots/gels are presented in Supplementary Figure 3. **b** The PI3K kinase activity was determined in NSCLC cells transfected with LY294002 was significantly lower than that transfected with mimic control, and the PI3K kinase activity in NSCLC transfected with both LY294002 and miR-425-5p mimic was significantly higher than that transfected with LY294002. Data: mean ± SD. * P < 0.05; ** P < 0.01; *** P < 0.001

It has been reported PTEN/PI3K/AKT signaling was closely related to cell proliferation and apoptosis [14–18]. Next, we explored the effect of miRNA-425-5p on PTEN/PI3K/AKT signaling. As shown in Fig. 5a, in comparison to NC groups, PTEN was down regulated in response to miR-425-5p mimics, whilst PI3K and p-AKT levels increased. In addition, NSCLC cells were treated with the PI3K/Akt inhibitor LY294002 or LY294002 + miR-425-5p mimics mimic (Fig. 5b), Kinase activity assays further showed that PI3K activity in NSCLC transfected with LY294002 was significantly lower than that transfected with mimic control (*P* < 0.01), and PI3K activity in NSCLC cells transfected with both LY294002 and miR-425-5p mimic was significantly higher than that transfected with LY294002 (P < 0.01). Take together; this implicates PTEN/PI3K/AKT signaling in the pro-tumorigenic effects of miR-425-5p.

## Discussion

The poor prognosis of LCa highlights the need for urgent therapeutic strategies. miRNAs are novel targets for cancer therapies and their dysregulation occurs in LCa tissue [19–22]. Duan et al. showed that miR-203 binds to ZEB2 to suppress EMT [23], Yuan et al. showed that miR-30a inhibits EYA2 migration and invasion [24], and Li et al. showed that miR-1304 inhibits LCa cell division through heme oxygenase-1 [25]. The cellular roles of miR-425-5p in LCa are poorly understood. In the present work, we further explore the underlying mechanisms of miR-425-5p-induced LCa cell progression.

In the present study, we confirmed that miR-425-5p is overexpressed in LCa cell lines and tissues implicating a role in LCa tumorigenesis. Upregulating miR-425-5p levels enhanced the cell survival and colony formation ability of LCa cells in vitro, implicating it as a novel LCa oncogene. In the mechanism, using the algorithms TargetScan website tools, we identified PTEN as the potential target of miR-425-5p. Furthermore, we performed Luciferase reporter assays and the results showed that miR-425-5p may directly target PTEN-3’UTR. The result of qRT-PCR and western blot also confirmed that over-expression of miR-425-5p could suppress the expression level of PTEN. All the above suggested that PTEN was a potential functional target of miR-425-5p. Moreover, MiR-425-5p also negatively related PTEN mRNA levels in LCa tissue. Rescue experiment indicated that exogenous PTEN expression inhibited the pro-cancer effects of miR-425-5p. PTEN was downregulated in LCa tissue. PTEN is a tumor suppressor with well-characterized phosphatase activity [26]. PI3K/AKT promotes cell cycle progression, inhibits apoptosis, and is known to be overactive in a multitude of human cancers [27, 28]. PTEN can suppress PI3K/AKT signaling and thus displays anti-cancer effects [29]. Upon assessment of the molecular mechanisms of miR-425-5p in LCa cells, its pro-cancer effects were found to be mediated through manipulation of the PTEN/PI3K/AKT signaling axis.

## Conclusion

In conclusion, we highlight miR-425-5p as an oncogene in LCa that promotes an oncogene phenotype by inhibiting PTEN. These findings enhance our knowledge of the role of miR-425-5p and reveal new therapeutic strategies for the diagnosis and treatment of LCa. Angiogenesis and metastasis biological experiments will clarify the functions and roles of miR-425-5p in LCa.

## Supplementary information

**Additional file 1: Supplement Figure S1.** Uncropped images of blots and gels related to Fig. 3. **Supplement Figure S2.** Uncropped images of blots and gels related to Fig. 4. **Supplement Figure S3.** Uncropped images of blots and gels related to Fig. 5. **Table S1.** The raw data of all colony formation experiments.

## Data Availability

The datasets used and/or analyzed during the current study are available from the corresponding author on reasonable request.

## Abbreviations

Lca: Lung cancer
PTEN: Gene of phosphate and tension homology deleted on chromsome ten
PI3K: Phosphatidylinositol 3-kinase
NSCLC: Non-small cell lung cancer
GCa: Gastric cancer
CRC: Colorectal cancer
HCC: Hepatocellular carcinoma
EMT: Epithelial-Mesenchymal Transition
