# Supplementary material for: miRNA-425-5p enhances lung cancer growth via the PTEN/PI3K/AKT signaling axis
Source: BMC Pulm Med. 2020 Aug 24;20:223. doi: 10.1186/s12890-020-01261-0 (PMC7447575; doi:10.1186/s12890-020-01261-0)
Supplement: Supplementary file 1 — Additional file 1: Supplement Figure S1. Uncropped images of blots and gels related to Fig. 3. Supplement Figure S2. Uncropped images of blots and gels related to Fig. 4. Supplement Figure S3. Uncropped images of blots and gels related to Fig. 5. Table S1. The raw data of all colony formation experiments. [file 12890_2020_1261_MOESM1_ESM.docx]

**miRNA****-425-5p enhances lung cancer growth via the** **PTEN/PI3K/AKT signaling axis**

Jin-shan Zhou, Ze-shan Yang, Si-yang Cheng, Jiang-hao Yu, Huang Juchao,Feng Qiang#

Cardiothoracic Surgery, The Fourth Affiliated Hospital，Zhejiang University School of Medicine, Yiwu, Zhejiang, 322000

Correspondence author: Feng Qiang Cardiothoracic Surgery, The Fourth Affiliated Hospital，Zhejiang University School of Medicine. Address: Shangchen Road NO.1 of YiWu, ZheJiang, China. Postal code:322000 Telephone：18867961036 email: kjhhev@163.com

**Supplement Figure S1**

Uncropped images of blots and gels related to Figure 3.

**
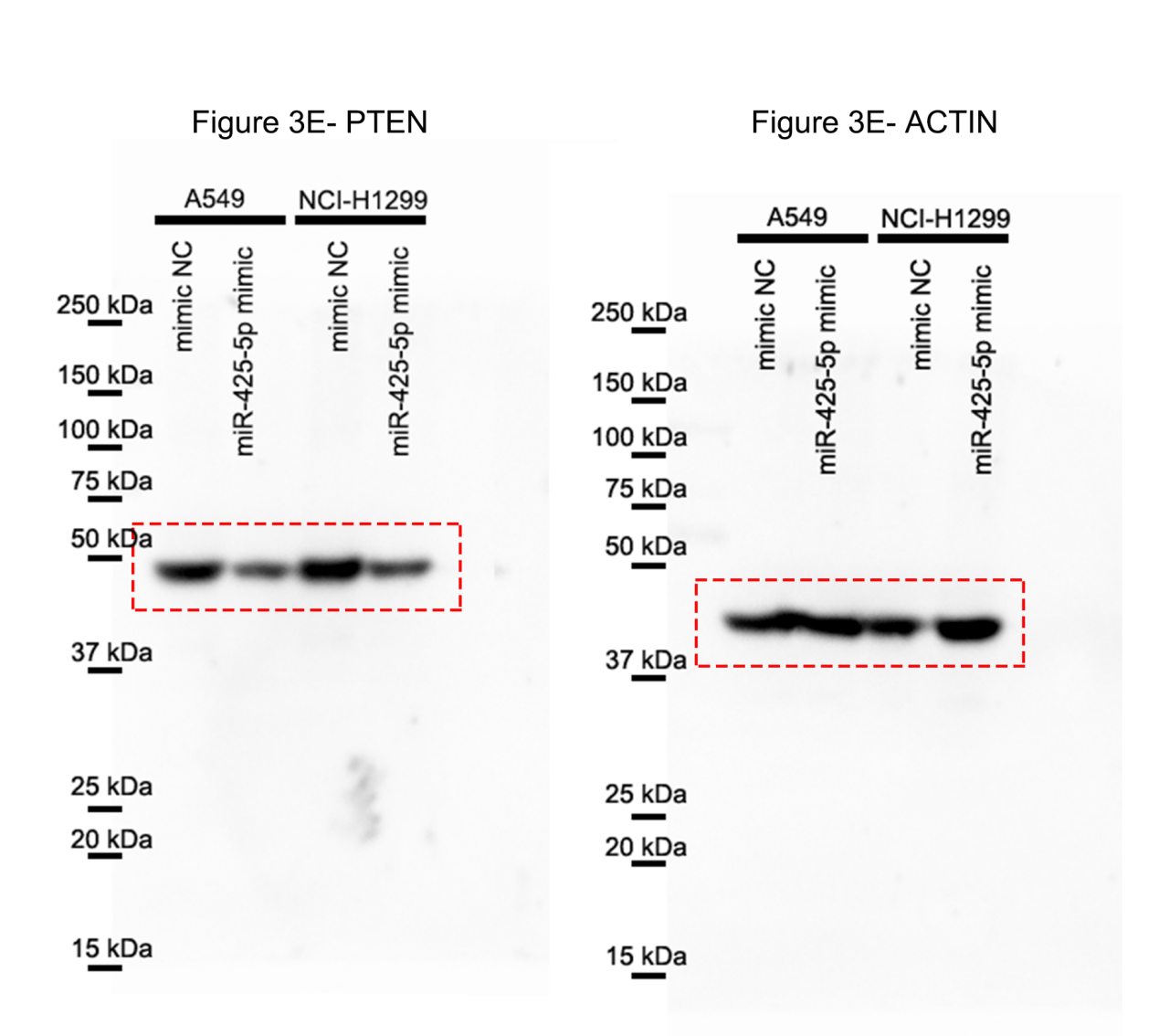
**

**Supplement Figure S2**

Uncropped images of blots and gels related to Figure 4.

**
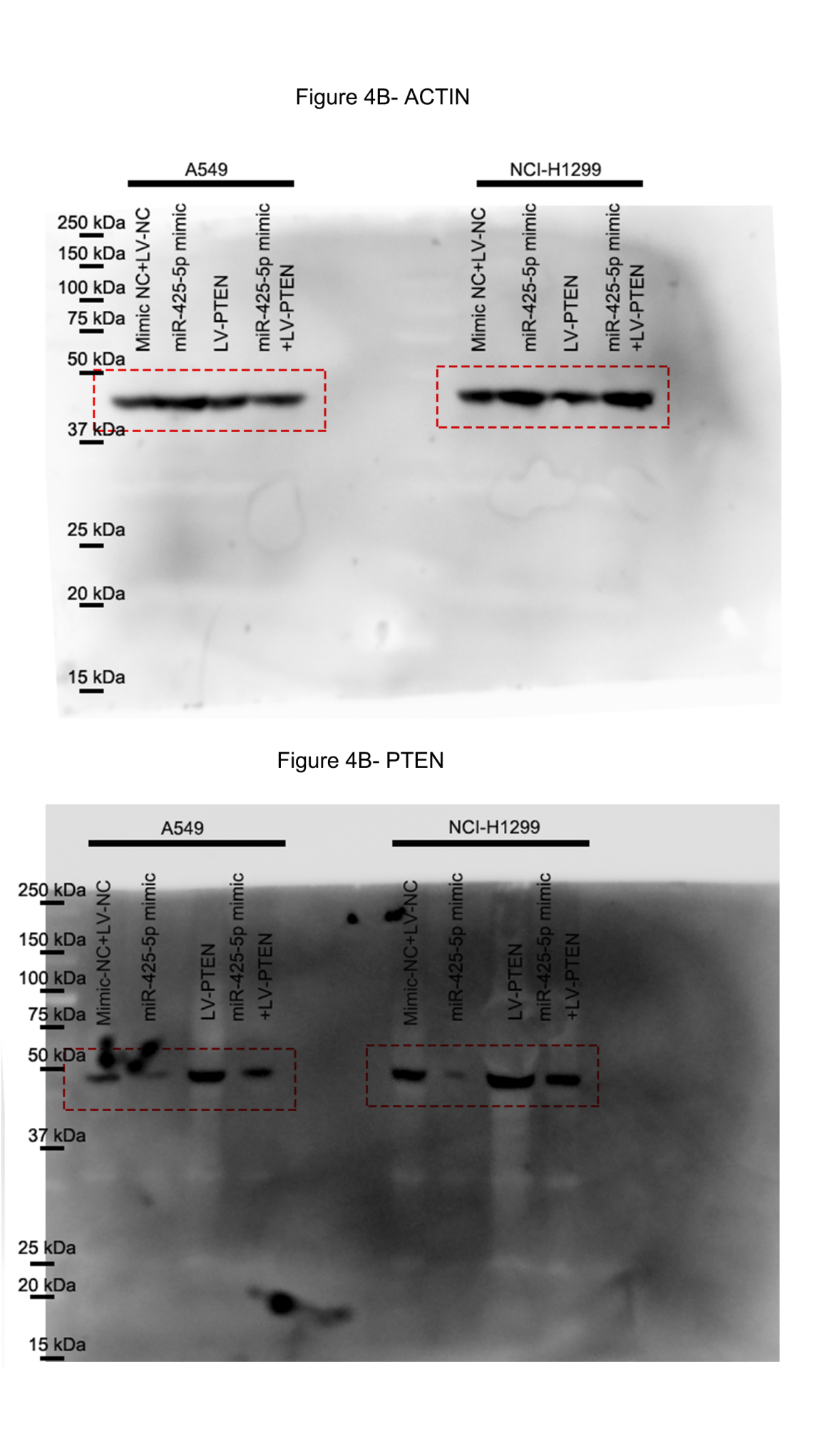
**

**Supplement Figure S3**

**
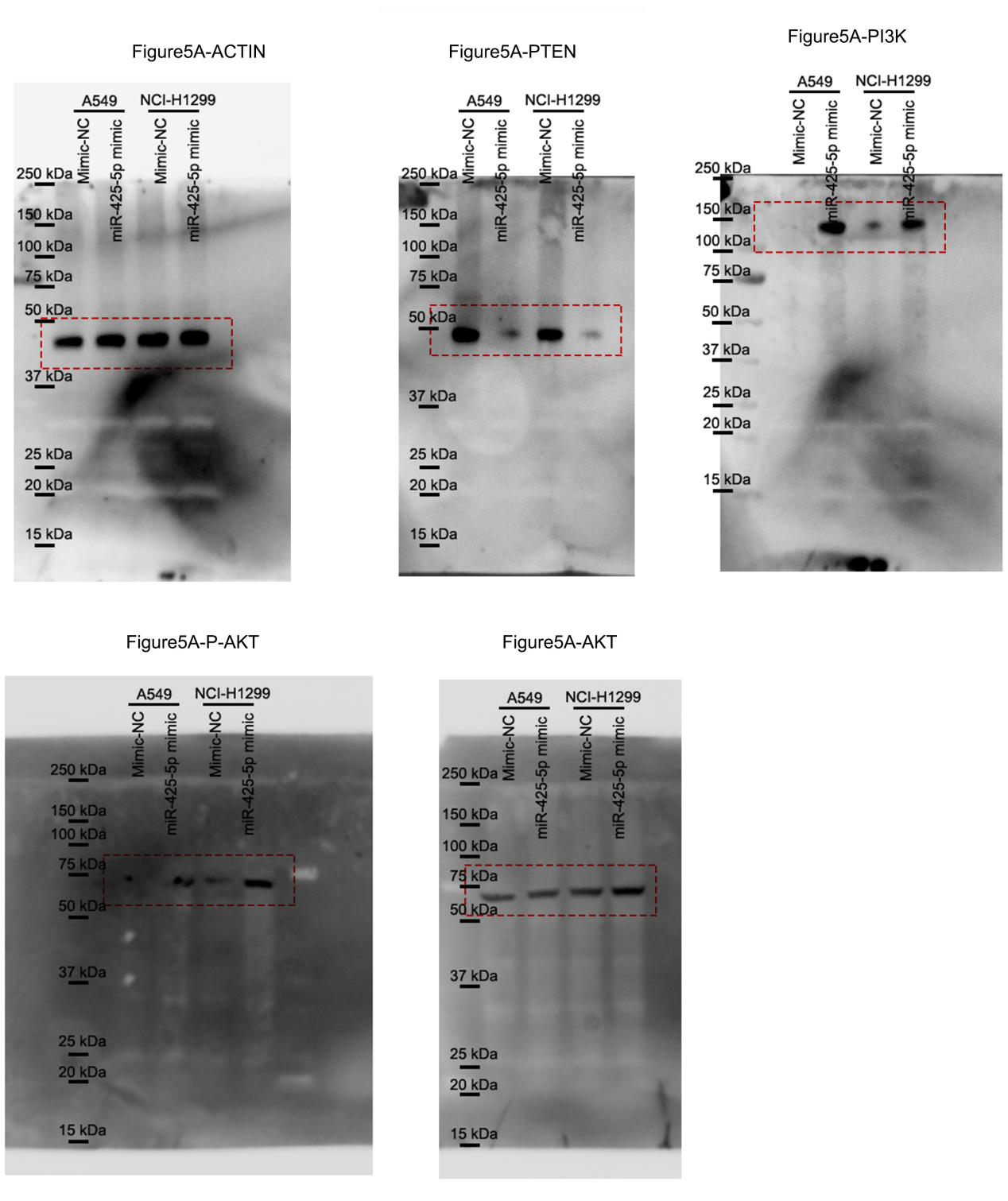
**Uncropped images of blots and gels related to Figure 5.

**Table S1 the raw data of all colony formation experiments**

| colony number | mimic NC | | | miR-425-5p mimic | | |
| --- | --- | --- | --- | --- | --- | --- |
|  | Repeat1 | Repeat2 | Repeat3 | Repeat1 | Repeat2 | Repeat3 |
| A549 | 332 | 350 | 310 | 169 | 145 | 165 |
| NCI-1299 | 410 | 430 | 390 | 210 | 198 | 225 |
